# Patterning Bacterial Communities on Epithelial Cells

**DOI:** 10.1371/journal.pone.0067165

**Published:** 2013-06-13

**Authors:** Mohammed Dwidar, Brendan M. Leung, Toshiyuki Yaguchi, Shuichi Takayama, Robert J. Mitchell

**Affiliations:** 1 School of Nano-Bioscience and Chemical Engineering, Ulsan National Institute of Science and Technology, Ulsan, Republic of Korea; 2 Nagoya Institute of Technology, Biomechanics Laboratory, Gokiso-cho, Showa-ku, Nagoya, Japan; 3 Department Biomedical Engineering and Macromolecular Science & Engineering Program, University of Michigan, Ann Arbor, United States of America; LAAS-CNRS, France

## Abstract

Micropatterning of bacteria using aqueous two phase system (ATPS) enables the localized culture and formation of physically separated bacterial communities on human epithelial cell sheets. This method was used to compare the effects of *Escherichia coli* strain MG1655 and an isogenic invasive counterpart that expresses the invasin (inv) gene from *Yersinia pseudotuberculosis* on the underlying epithelial cell layer. Large portions of the cell layer beneath the invasive strain were killed or detached while the non-invasive *E. coli* had no apparent effect on the epithelial cell layer over a 24 h observation period. In addition, simultaneous testing of the localized effects of three different bacterial species; *E. coli* MG1655, *Shigella boydii* KACC 10792 and *Pseudomonas* sp DSM 50906 on an epithelial cell layer is also demonstrated. The paper further shows the ability to use a bacterial predator, *Bdellovibrio* *bacteriovorus* HD 100, to selectively remove the *E. coli*, *S.* boydii and *P*. sp communities from this bacteria-patterned epithelial cell layer. Importantly, predation and removal of the *P. Sp* was critical for maintaining viability of the underlying epithelial cells. Although this paper focuses on a few specific cell types, the technique should be broadly applicable to understand a variety of bacteria-epithelial cell interactions.

## Introduction

In the human body, the interactions occurring between the epithelial cells and bacteria are diverse and complicated, ranging from commensalism to invasion and parasitism [1]. Attempts to study these interactions and their mechanisms in depth have been made with the goal being improved infection prevention and treatment options. In addition, a better understanding of bacteria-epithelial cell interactions may also broaden the use of bacteria as therapeutic agents to eliminate tumor cells [2] or as carriers for therapeutic agents into epithelial cells [3,4].

In conventional cell culture formats currently available in labs it is difficult to co-culture epithelial cells with bacteria in the same medium and study their interactions because the bacterial cells are freely suspended and may not associate with the epithelial cells. Furthermore, the bacterial cells tend to overgrow in the nutrient rich and favorable temperatures of epithelial cell culture conditions. This causes rapid nutrient depletion and dramatic changes in pH, thus rendering the medium unsuitable for growth of the epithelial cells [5]. To circumvent this problem, exposures between epithelial cell cultures and the bacteria are usually kept short, ranging from minutes to hours, followed by the removal of the bacteria shortly thereafter [2,6,7]. Research groups have also generated dynamic co-cultures using a flow-through continuous culturing system [8,9] or microfluidic devices, such as the one used by Jayaraman’s group to study the role of commensal bacteria in preventing pathogen infection in the gastrointestinal tract [5] or that developed by Fan Yuan’s group to study the bacterial proliferation in the three dimensional structure of a tumor [10].

Recently, our group reported on the aqueous two phase system (ATPS) patterning of different bacterial strains in a single Petri dish to form suspended “colonies” that are physically separated but chemically connected through the aqueous phase [11,12]. In a separate study, we expanded this technique to develop localized and patterned biofilms and subsequently used these biofilms to study interactions between different bacterial strains [13]. In this paper, the benefits of ATPS confinement are extended to applications where bacteria are patterned within a restricted aqueous space over epithelial cells for prolonged periods, i.e., 24 h, without release of the bacteria into the surrounding growth media. To illustrate this, experiments were performed using recombinant *E. coli* expressing the invasin gene from *Y. pseudotuberculosis* as well as with a panel of bacterial strains. Furthermore, developing static localized bacterial communities on epithelial cells can have other applications to facilitate studying the interactions between epithelial cells and various bacteria. 

## Results

### Bacteria can be patterned over epithelial cells using aqueous two phase system micropatterning to give localized bacterial communities

To test the possibility of using ATPS technology to pattern bacteria on epithelial cell monolayers, we first compared three different ATPS formulations (Table 1) with regards to their effects on a confluent layer of human mammary epithelial cells (MCF 10a). Two of these formulations were used before for printing mammalian cells [14] while the third was a more viscous formulation used in a recent study for patterning bacterial cells and biofilms on polystyrene or PDMS surfaces [13]. MCF 10a cell monolayers were covered with the DMEM/F12-infused polyethylene glycol (PEG) rich phase of each of the three formulations. The plates were then incubated for 24 h to investigate the effects of the different PEG solutions on epithelial cells viability (Figure S1). Both the first and the second formulations had no adverse effects. This is in contrast to the third formulation which showed a significantly higher number of dead epithelial cells, as evidenced by EthD-1 staining (Figure S1).

**Table 1:** The composition of the three ATPS formulations tested in this study.

**ATPS formulation tested**		**DEX M.W.**		**DEX Conc. (w/w)**		**PEG M.W.**		**PEG Conc. (w/w)**	
1st formulation		500,000		3.2%		35,000		2.5%	
2nd formulation		500,000		5%		8,000		4%	
3rd formulation		20,000		7%		200,000		7%	

**Figure 1:**
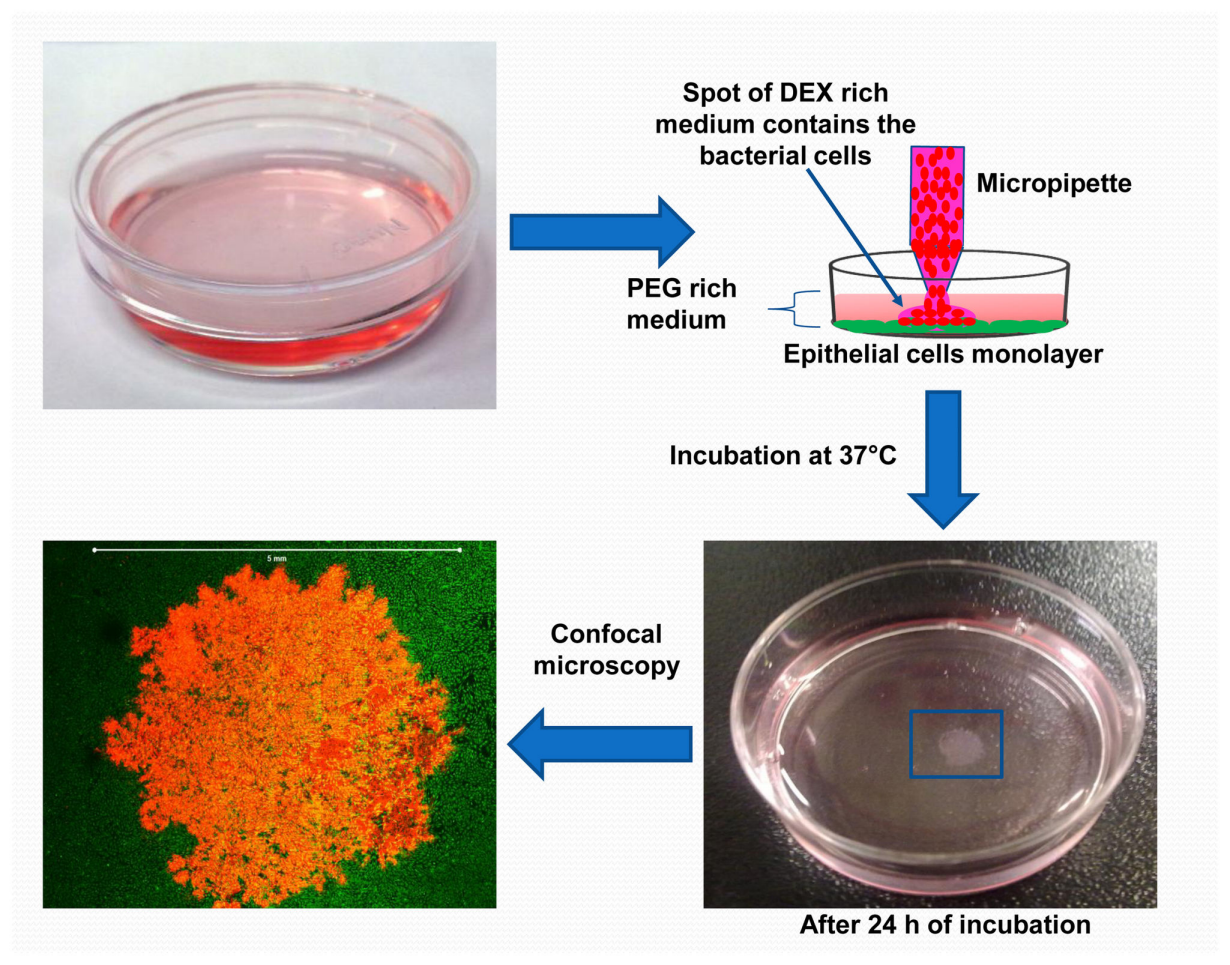
A schematic diagram shows the procedure of making ATPS derived bacterial communities on epithelial monolayer using the first ATPS formulation. The mammalian cells (MCF10a cell line) were grown in supplemented DMEM/F12 medium inside a 35 mm Petri dish as usual until it formed a monolayer sheet (~80% confluency). The growth medium was then removed and replaced with 2.5 ml of PEG rich medium. This is followed by spotting drops of the DEX rich medium containing the bacteria (*E. coli*/pHKT3) using a conventional micropipette. After 24 h of incubation at 37°C, the resulting bacterial community is visible and can be analyzed microscopically (scale bar: 5 mm).

Consequently, the first and the second formulations were used in experiments to pattern a commensal non-pathogenic bacterium, i.e., *E. coli* MG1655, over an MCF 10a monolayer. For these experiments, the bacterial cells were re-suspended in the dextran (DEX) rich phase of each formulation and spotted on MCF 10a monolayers within the corresponding PEG rich phase. The schematic for this process is shown in Figure 1. As shown in Panel D of Figure S1, after the medium was removed and the plate washed as described in the Materials and Methods, a clear bacterial community associated with the MCF 10a monolayer was clearly observable and illustrates that stable, round bacterial communities can be generated with the first formulation. In contrast, for the second formulation, although it could also isolate the bacteria inside the DEX droplet, the bacterial community formed was not attaching well to the underlying epithelial layer and most of it was detached when the plate was washed (Figure S1, Panel D). Based on these results, the first ATPS formulation, which has the lowest polymer concentrations and interfacial tension between the two phases (0.01 mJ/m_2_ as determined previously [14]), was the most successful when patterning bacteria on epithelial cells. Consequently, this formulation was used in all subsequent experiments.

**Figure 2:**
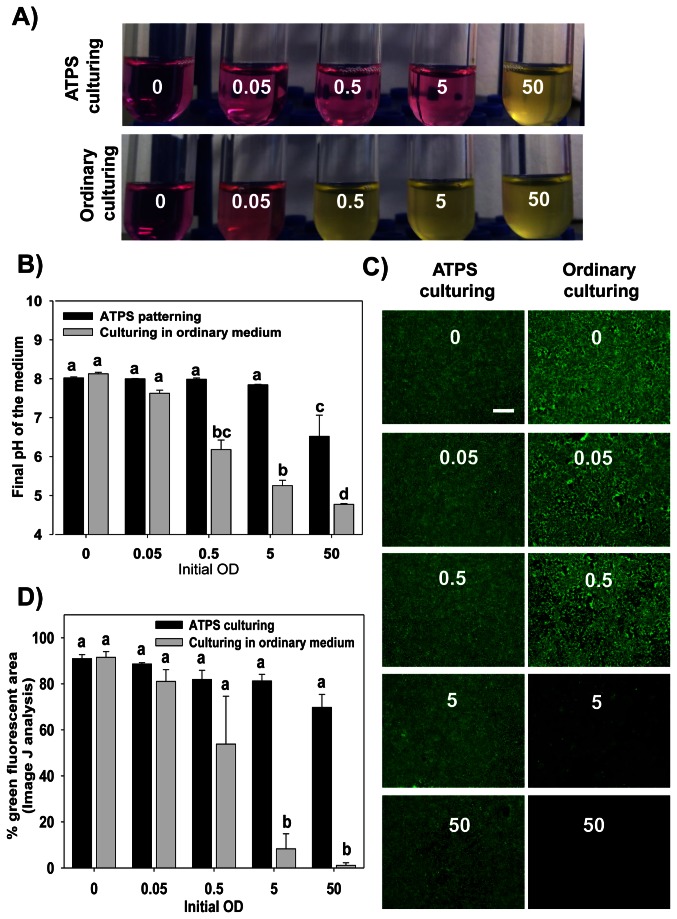
Comparing ATPS culturing with the ordinary culturing of bacteria on MCF 10a layer substrate. *E. coli* MG1655 was spotted on an MCF 10a monolayer at different rOD values using both ordinary culturing and ATPS technique. For the former, the bacteria were re-suspended into ordinary DMEM/F12 medium at the required rOD and then spotted directly as single 0.3 µl droplet into 2.5 ml of the medium. For the ATPS patterning, the bacteria were re-suspended in DEX rich DMEM/F12 medium and one 0.3 µl droplet was spotted in 2.5 ml of PEG rich medium of the first ATPS formulation. All plates were then incubated at 37°C for 24 h before being analyzed. A) The change in the color of the medium (with phenol red indicator) 24 h after spotting different doses of *E. coli* inside 35 mm Petri dishes. The medium was aspirated from the dishes in 10 ml glass tubes as shown. B) The average pH values for the media shown in panel A with the error bars representing the standard errors of 3 replicates for each case. Statistical analysis was performed using ANOVA followed by Tukey post hoc test (a, b, c and d = *p* < 0.05). C) Representative photos for the MCF 10a sheet in the Petri dishes after 24 h of incubation with the bacteria for both types of culturing. The medium was aspirated, and the plates were washed well to remove the attached bacteria. Afterwards, the epithelial cell sheet was stained with Calcein AM dye and observed using an epiflourescence microscope. Scale bar: 500 µm. D) Plot showing ImageJ analysis for epithelial areas fluorescing green in the photos shown in panel (C). The error bars represent the standard errors of three images for each case (a, and b = *p* < 0.05).

To further demonstrate the efficacy of using this ATPS technique to isolate the bacterial cells within a defined locale and limit their unconfined spread, we performed localized dispensing of suspensions of the same strain (*E. coli* MG1655) at different initial rOD (reconstituted optical densities) values using both ordinary culturing and ATPS technique. For the former, the bacteria were re-suspended into ordinary DMEM/F12 medium at the required initial rOD and then spotted directly as a 0.3 µl droplet into 2.5 ml of ordinary DMEM/F12 medium. For the ATPS patterning, the bacteria were re-suspended in DEX rich DMEM/F12 medium and spotted as 0.3 µl droplets in 2.5 ml of PEG rich DMEM/F12 medium using a conventional micropipette as mentioned before [11]. This experiment was done in 35 mm Petri dishes with no epithelial cells (polystyrene surface only) (Figure S2) as well as on an epithelial cell monolayer (Figure 2). After incubation for 24 h, the medium, including the freely suspended and unattached bacterial cells, was aspirated into 10 ml glass tubes and the pH of each was measured.

As shown in Figure 1, the ATPS patterning technique effectively isolated the bacterial culture and limited the bacterial growth to only a small area as defined by the original spot, thereby preventing them from spreading out into the medium. Figure 2 demonstrates that the pH of the overall medium was stable regardless of the initial rOD spotted, up to a rOD of 5 (Figure 2A and B). Tests performed in parallel without MCF 10a cells show that the drop in pH is partially attributable to the metabolic activity of the epithelial cells (Figure S2). However, a similar trend was seen where the pHs within the ATPS-spotted cultures were only slightly decreased for the rODs tested while those of the unmodified media decreased as the rOD increased. The efficacy of ATPS spotting on epithelial cells was limited with higher cell densities, however, as a rOD of 50 led to both a significant drop in the pH as well as a visible change in the medium color due to the presence of phenol red as a pH indicator (Figure 2B). This likely resulted from excessive growth of the bacterial cells spilled outside of the dextran droplet as the culture medium was turbid.

The localized containment of the bacteria within the ATPS-droplet led to better survival of the MFC 10a cells during this 24 h exposure, as evidenced in Figure 2C, and D. This figure shows that the MFC 10a cell viability, morphology and attachment were not affected as long as the bacterial culture was contained inside the DEX droplet. In contrast, the images from samples performed using the unmodified media show a gradual loss in MFC 10a confluency and attachment as the initial rOD was increased.

Here, it is also worthy to note that attempts to spot the bacteria at a rOD of 0.5 using either a DEX-based droplet in ordinary DMEM/F12 media, i.e., without PEG added, or within a DMEM/F12 droplet, i.e., without DEX added, inside PEG rich DMEM/F12 media were unsuccessful. In both cases, the bacteria spread throughout the media and the resulting pH after 24 h was 5.8 ± 0.6, demonstrating the needed for both the DEX and PEG phases for this technique to effectively localize the bacteria within the media.

### The size of the bacterial spot can be controlled by changing the initial volume of the DEX droplet

**Figure 3:**
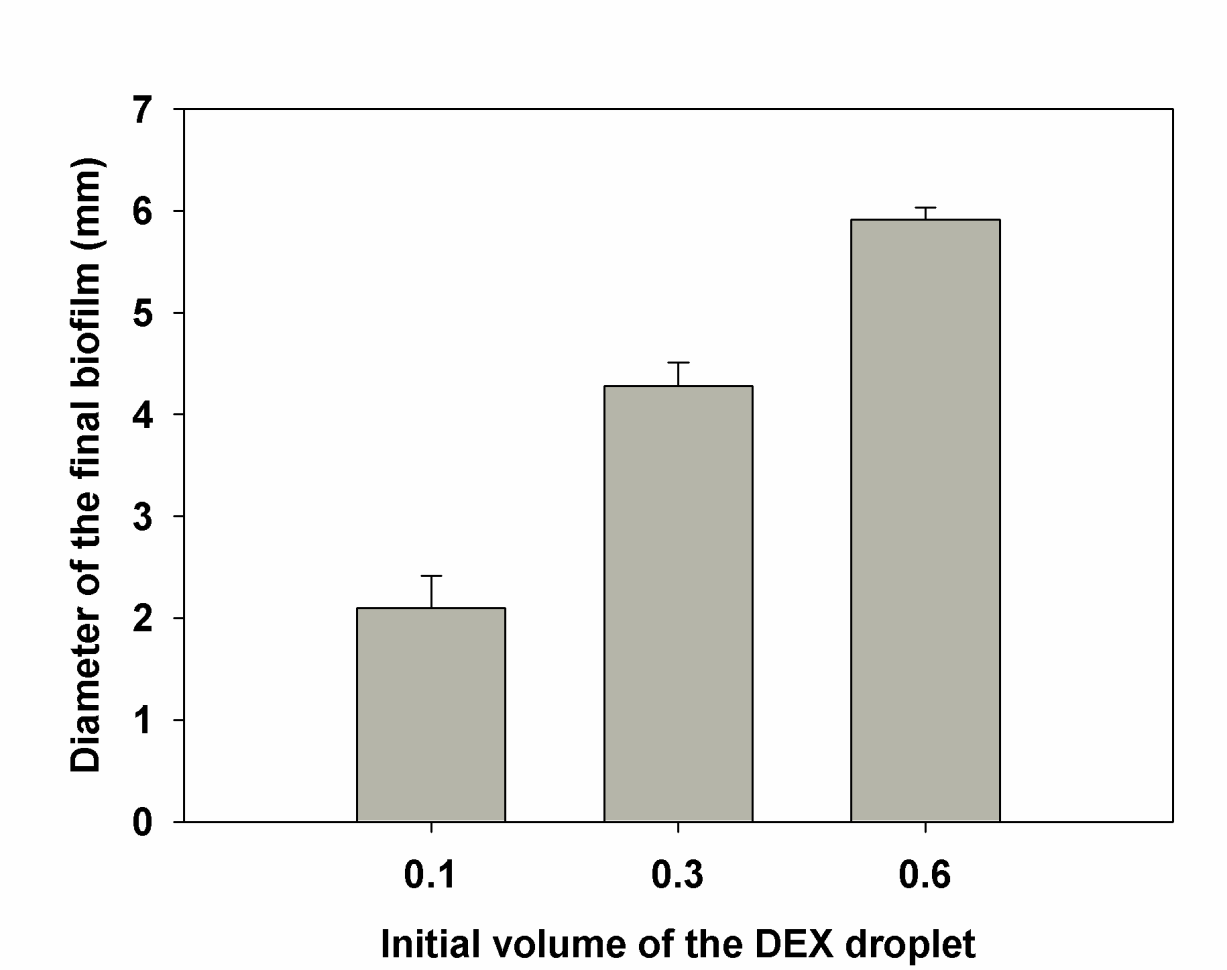
The area of the final spot can be controlled through changing the initial DEX droplet volume. As shown in the figure, by changing the initial volume of the DEX-rich droplet, the area of the final spot made using *E. coli* MG1655 on the epithelial cell layer can be precisely controlled. Measurement of the spot area was done after 24 h of incubation. The values are the ave**r**age areas from three droplets for each volume with the error bars representing the standard error.

In a previous study done by our group [13], we showed that the final size of the DEX spot and, hence, the community formed can be controlled by adjusting the initial volume of the droplet. Here, in this study, this same principle was expanded upon and applied to develop isolated bacterial communities on an epithelial cell layer (Figure 3). Owing to the difference in the surface chemistry and also interfacial tension of the ATPS formulation used in this study, the spots formed are somewhat larger than those reported previously, with diameters ranging from 2 to 6 mm when 0.1 to 0.6 µl of the culture was spotted, respectively.

### Evaluating the effects of virulence gene expression using isogenic bacterial strains growing on a single epithelial monolayer

**Figure 4:**
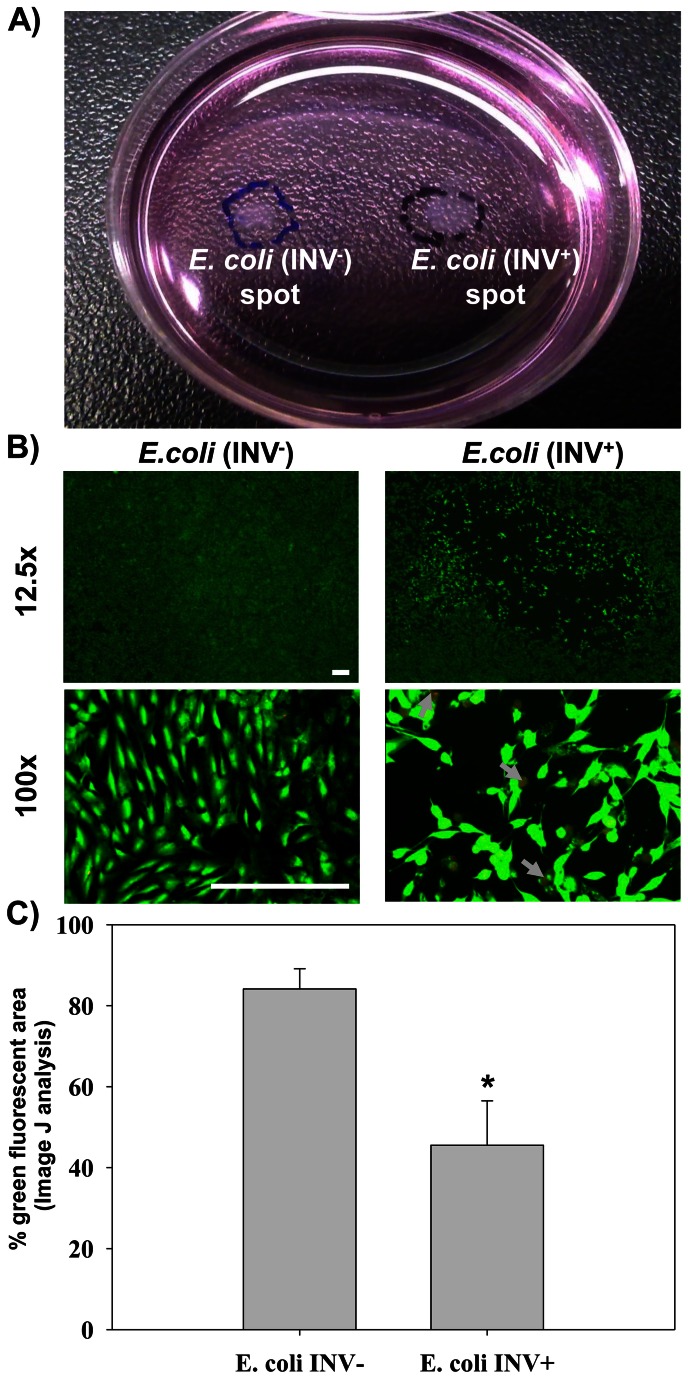
Investigating the effect of invasin on epithelial cells. A) *E. coli* MG1655/pLacCherry (INV^-^) and its isogenic counterpart, *E. coli* MG1655/pINVCherry (INV^+^) were spotted on epithelial cells monolayer in the same plate using ATPS technique. The photo shows that the two spots are visible to the naked eye after 24 h of incubation. B) Microscopical analysis of the epithelial cell layer beneath the two spots (INV^-^ and INV^+^). After washing out the two bacterial communities, the epithelial cells were stained with Calcein AM dye to compare the effects of the two bacterial spots on the viability of the underlying epithelial area. The photos were taken using an epifluorescence microscope at two different magnifications as shown. The white arrows point to the *E. coli*-invaded epithelial cells. Scale bar: 200 µm. C) Plot showing the integrity of the epithelial cells sheet underneath both spots. The fraction of the epithelial area fluorescing green underneath both spots were calculated using ImageJ program. The error bars represent the standard error of 4 spots for each case. Statistical analysis was performed using Student t-test. * *p* < 0.05.

To demonstrate the applicability of this method to study bacterial interactions with epithelial cells, we next assessed the effects that expression of a known virulence factor had on the viability of an underlying MFC 10a culture. For this, we used *E. coli* MG1655/pLacCherry (*inv* ^-^) and its isogenic counterpart, *E. coli* MG1655/pINVCherry (*inv* ^+^), which expresses the invasin gene from *Y. pseudotuberculosis*. The expression of the *inv* gene allows *E. coli* to readily invade MFC 10a cells by binding to β1 integrin on the epithelial cell surface [15]. For these tests, both *E. coli* strains were patterned side by side on an MCF 10a monolayer in the same culture dish (Figure 4).

The plate was incubated for 24 h to allow the bacterial communities to grow and interact with the underlying epithelial layer. Afterwards, the plate was washed well to remove the attached bacterial community but without detaching the epithelial cells. As shown in Figure 4B, the MFC 10a cells beneath the *E. coli* (*inv* ^-^) spot were viable and were generally unaffected by the prolonged exposure to this strain. In contrast, the invasive *E. coli* (*inv* ^+^) led to pronounced destruction and detachment of the underlying epithelial cells. Analyzing the images using the ImageJ program found that the area of the plate covered by epithelial cells beneath the two spots decreased from 84% for the *inv* ^-^ spot to about 46% for *inv* ^+^ (Figure 4C). Under higher magnification, it was clear that a large number of epithelial cells remaining underneath the invasive *E. coli* spot were extensively invaded by this bacterial strain. This is in contrast to those underneath the *E. coli* MG1655/pLacCherry spot, which showed only few bacteria associated with the MFC 10a cells.

### Patterning different species of bacteria on epithelial cells in the same plate

**Figure 5:**
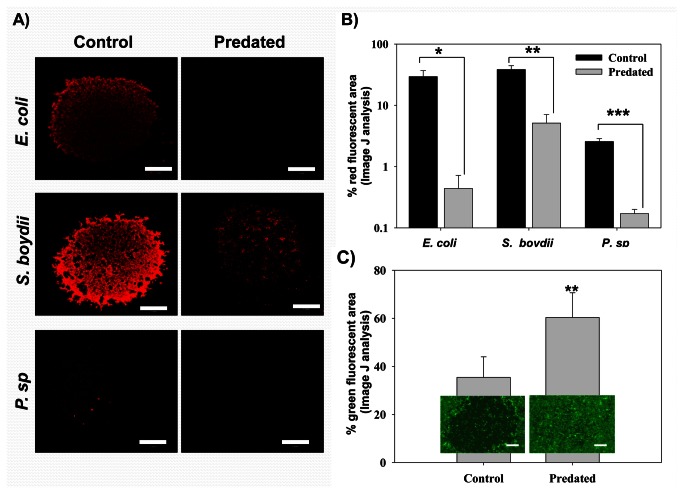
*B* *. bacteriovorus* predation of *E. coli*, *S.* boydii, and *P*. sp communities on MCF 10a monolayer. The three preys; *E. coli* MG1655, *S. boydii* KACC 10792, and *P*. sp DSM 50906 were suspended in the DEX rich phase and spotted as separate drops in the same plate at an initial rOD of 0.5. For the predator plates, *B* *. bacteriovorus* HD 100 was added to the PEG phase as described in the Materials and Methods section. The control plates were filled with only the PEG rich phase, i.e., without the predator added. Both sets of plates were incubated for 24 h then observed microscopically. A) The confocal microscopy photos for the three preys communities in both the control plate and the predated ( *B* *. bacteriovorus* HD 100 containing) one. All images in panels A were taken using the same microscopy settings so that the data for the three organisms could be compared. Scale bar: 1mm. *B*) ImageJ analysis for the images in panel (A) showing the percent area of the image fluorescing red. The error bars show the standard error obtained from the analysis of at least 3 images for each case. Statistical analysis was performed using t-test. * p < 0.05, ** *p* < 0.01, *** *p* < 0.001. C) *B* *. bacteriovorus* predation protected MCF 10a against the effects of *P*. sp DSM 50906. *P*. sp was spotted at a rOD of 5. After incubating the plates for 24 h at 37°C, the epithelial cells were stained and observed using an epifluorescence microscope. The plot compares the green fluorescence of the epithelial areas underneath the *P*. sp spots for both the control and predated plates. The error bars represent the standard error of six replicates for each case. ** p < 0.01. The images are representative photos showing the appearance of the two areas as observed using epiflourescence microscope. Scale bar: 500µm.

The above results clearly demonstrate that mutants of one bacterial strain can be patterned within a single culture dish and, as such, can be extrapolated to different species of bacteria. This was demonstrated in Figure 5, and Figure S3 where red fluorescing *E. coli* MG1655, *Shigella boydii* KACC 10792 and *Pseudomonas* sp. DSM 50906 were successfully patterned as individual spots within a single culture of MFC 10a epithelial cells.

After 24 h, the three strains formed separated and well defined bacterial communities which corresponded to the footprints of the DEX droplets (Figure S3). It was noticed, however, that the *S. boydii* cells were not attaching tightly to the underlying epithelial cell layer. When stained with Calcein AM and observed under fluorescent microscopy, the MCF 10a layer beneath the *E. coli* spot appeared viable and was visually indistinguishable from the surrounding epithelial areas that were not exposed to the bacteria. The same was also true for the *S. boydii* spot, suggesting that this strain used here is exceptionally avirulent [16] (Figure S4). In contrast, for the spot containing P. sp., there was significant damage to and detachment of the underlying epithelial cell layer. This was surprising as this strain grew at a much slower rate and was much less proliferating when compared to the other two strains. In fact, the P. sp. spot could be seen clearly only when using fluorescent microscopy, which is in contrast to the other two strains whose spots were clearly visible to the naked eye (Figure S3).

To study this further, *P*. sp DSM 50906 was patterned in a subsequent experiment at different rOD values (Figure S5). It was immediately clear that the degree of damage to the epithelial cell layer was dependent upon the initial seeding dose within the DEX droplet, with a rOD of 5 and 0.5 leading to the greatest amount of damage and detachment while the lower rODs tested showed less by comparison.

### Predation of P. sp DSM 50906 by *Bdellovibrio* *bacteriovorus* HD 100 protects the epithelial cells

*Pseudomonas* sp. DSM 50906 is a well known prey bacterium for the predatory strain *Bdellovibrio* *bacteriovorus* HD 100 [17]. This predator has been previously shown to predate upon and reduce the population of many human pathogens [18] and is regarded as a potential probiotic or alternative to chemical antibiotics [19,20]. Consequently, tests were performed to assess if this predatory strain could protect the epithelial cell layer from P. sp. DSM 50906 when spotted at a rOD of 0.5. For comparison, tests were also performed with *E. coli* and *S. boydii* within the same MFC 10a culture plate. To do this, the predator was suspended in the PEG rich DMEM/F12 medium and then added to the MCF 10a culture plate. For the control plate, only PEG rich DMEM/F12 medium was added without the predator. The three preys were suspended in the DEX rich phase and spotted as 0.3 µl droplets as usual and the plates were incubated for 24 h.

The reduction in both the bacterial biomass and its fluorescence (Figure 5A and B) clearly show that the communities formed by each of the three test strains were significantly reduced in the presence of *B* *. bacteriovorus* HD 100. ImageJ analysis found that the *E. coli* fluorescence was reduced by more than 60-fold, while those of *S. boydii* and *P*. sp. were reduced by about 8- and 15-fold, respectively. Furthermore, in tests performed with P. sp. DSM 50906 at a rOD of 5, damage to the MFC 10a cell layer was significantly reduced when the predator was added to the plate. As shown in Figure 5C, the area covered by the epithelial cells underneath the P. sp spots increased from 35% for the control plate to 60% for the plate having the predator added (Figure 5c), indicating that predation by *B* *. bacteriovorus* HD 100 was capable of protecting the underlying epithelial cells.

## Discussion

In this study, we demonstrated how the use of ATPS technology [21] can be extended to pattern bacteria on epithelial cells and provided several examples of its application. Using an ATPS formulation published previously to print mammalian cells [14], we were able to pattern several different bacterial species on an MCF 10a epithelial monolayer. Once spotted, the bacteria are confined within the DEX-rich spots due to thermodynamically stable partitioning preferences. Consequently, tests can be performed for an extended period of time (at least 24 h), which is in contrast to previous studies where the exposure was limited to only a few hours. Furthermore, given a sufficient amount of time, these bacterial spot cultures form patterned communities which adhered well to the underlying cell layer and were not washed away upon removing the medium or gentle washing of the plate suggesting that these formed communities started to develop biofilms.

This study found that the size of these spots can be precisely controlled by simply changing the initial volume of the DEX suspension spotted in the PEG phase, with the smallest having a diameter of only 2 mm. Although not tested here, based upon the results of this and previous studies [11–14,21–24], it is reasonable to expect that this method can be extended for use with a variety of eukaryotic cell types and bacteria, both pathogenic and commensal ones.

Subsequent tests were performed using a fluorescent *E. coli* strain and an isogenic variant of this strain that additionally expresses the invasin gene from *Y. pseudotuberculosis*. The results revealed that the epithelial layer beneath the invasive *E. coli* was extensively damaged while that beneath the *inv* ^-^ strain was viable. Furthermore, many of the epithelial cells exposed to the invasive *E. coli* strain showed bacteria growing intracellularly but only few non-invasive *E. coli* were seen associated with MFC10a cells. Although this is not unexpected based upon other studies where *E. coli* also expressed the *inv* gene [25], this study used the activity of the invasin protein to illustrate the capability of our co-culture system to evaluate the effects, individual virulence factors have, on the viability of the underlying cell layer. One of the unique features of our co-culture system is that both the control and isogenic strains can be evaluated side-by-side without concern that the bacterial droplets will mix or that the strains will escape into the surrounding media. Since this can be accomplished in a contiguous fashion within a single epithelial cell culture, this will both minimize the need for additional resources as well as make the comparison more reliable.

This concept was expanded further in tests where three different bacterial species, including *S. boydii* and a *Pseudomonas* strain, were spotted to form separate communities and their individual effects on the underlying epithelial cells assessed independently but within a single culture. Two of these communities, *E. coli* MG1655 and *S. boydii* KACC 10792, did not have a profound effect on the epithelial cells. The absence of any damage with *E. coli* was not surprising, but a similar result with *S. boydii* suggests that this strain used in our study was avirulent. Previous studies have shown that the virulence of *Shigella* strains is dependent upon the presence of a large plasmid [26] and that these strains can become avirulent when this plasmid is deleted or mutated [27]. It would appear that our strain has lost its ability to invade epithelial cells. This may also explain the weak binding seen in this study as a related strain, *S. flexneri*, was found to be less adherent to epithelial cells when became avirulent [28].

In contrast to *E. coli* MG1655 and *S. boydii* KACC 10792, *P*. sp DSM 50906 was clearly lethal towards MFC10a as evidenced by the damage to and loss of integrity of the epithelial cells beneath its spot. Furthermore, this effect was density-dependent as a rOD of 0.5 led to the greatest loss in cell viability and attachment, while lower rODs showed mitigated responses in comparison. This finding demonstrated another benefit of using ATPS spotting, namely a strict control over the multiplicity of infection and the ability to evaluate bacterial density effects.

As the DEX-rich droplet extends over the epithelial cells, the bacteria trapped within by stable partitioning are localized to near the epithelial cell layer, as opposed to throughout the media as found in traditional bulk phase culture. The result is an increase in association between the bacteria and the epithelial cell layer, which is important when studying host–pathogen interactions. For example, some bacteria need large infective doses to cause an infection, such as *Salmonella* , while other pathogens need only small doses, like enterohaemorrhagic *E. coli* (EHEC), which can cause an infection with as few as 10 cells [29]. Consequently, based upon the results of this study, it should be readily possible to study and evaluate the dose-dependent effects of virulent strains on pathogenesis within tissue culture plates.

We believe that the capability of co-patterning different strains of bacteria in the same plate will be a useful tool in building bacterial communities and studying their effects on epithelial cells. It is also a powerful platform for studying the chemical interaction and communication between physically isolated bacterial colonies and how this interaction may alter the virulence of the pathogenic strains. For example, Jayaraman’s group reported previously about the differential effects, indole, epinephrine, and norepinephrine have, on the virulence, chemotaxis and motility of EHEC [30]. Also, in 2003, Sperandio et al. [31], reported on the role of quorum sensing in development of EHEC infection, and how pathogens need special quorum sensing signals (AI3) produced by commensal *E. coli* in the intestine to recognize the intestinal cells and start the infection. Likewise, various bacterial strains produce the quorum sensing molecule AI-2, which is known to affect biofilm formation, adherence to eukaryotic cells and motility [32,33]. Interactions like these between different bacterial strains on epithelial cells surface would be better studied using this technique, and the results may help us extend our understanding about the progression of infection *in vivo*.

Finally, we showed how the bacterial predator–prey interaction can be studied on epithelial cell cultures using this technique. *B* *. bacteriovorus* HD 100 is a predatory bacterium which lives by invading other Gram-negative bacteria and lysing them from inside [17]. Numerous studies have shown that this predator is not pathogenic to higher organisms [34–36] and that it has a significantly low immunogenicity [37]. Furthermore, *B* *. bacteriovorus* HD 100 has a broad prey range, which includes many human and animals pathogens [18]. Therefore several recent reports have mentioned the potential application of *B* *. bacteriovorus* HD 100 and similar strains as a probiotic or living antibiotic [19,20]

Although several studies have established the ability of *B* *. bacteriovorus* HD 100 and similar predators to eradicate harmful planktonic bacteria [38] and remove preformed biofilms from surfaces [39–41], the results of this study show how this predator can be used as a preventative measure to effectively control planktonic pathogens and prevent them from establishing communities on epithelial cell surfaces. Moreover, as noted above, *B* *. bacteriovorus* HD 100 is regarded as a potential probiotic [19] and this study provides *in vitro* evidence for such capability as this predatory bacterium was able to protect the MCF10a epithelial cells from the damaging effects of P. sp, DSM 50906. To the best of our knowledge, this is the first study which shows the ability of *B* *. bacteriovorus* HD 100 to attack and prevent pathogens from establishing their communities on epithelial cells surfaces.

## Conclusions

We are describing here the application of ATPS technique for patterning bacterial species on epithelial cell layers in small Petri dishes. Using this technique, we could successfully form localized bacterial communities and limit the bacterial infections to specified areas on a confluent layer of epithelial cells inside the Petri dish. The diameter of the resulting bacterial community can be as small as 2 mm with possibility of producing even smaller ones using optimized protocols. The technique may see a wide range of applications, from comparing the properties of one strain with its own mutant in the same Petri-dish and on the same epithelial cells substrate, to patterning different bacterial species on epithelial cells and studying the interactions between these bacterial communities chemically without being physically in contact and how this can affect their virulence or their ability to invade the epithelial cells. We also showed how we can use this ATPS technology to study the ability of bacterial predators to predate on different pathogenic bacteria preventing them from establishing their communities on epithelial cells surfaces and hence protecting the epithelial cells from the toxic effects of these pathogens. We believe that the applications of this technique can be extended even further and it can be a useful tool to help us understand more about bacterial-bacterial, and bacterial-epithelial interactions. 

## Materials and Methods

### Epithelial cell Culture and Monolayer Seeding

Human mammary epithelial cells line, MCF10a, was obtained from ATCC (cat# CRL-10317). Cells were kept as frozen stocks at -160°C. The cells were inoculated in cell culture flasks (25 cm^2^) (SPL Life Sciences, Korea) at a concentration of 5 x 10^5^ cells/flask and incubated at 37°C and 5% CO_2_. The media used was reported previously [42]. Briefly, DMEM/F12 medium (Invitrogen) was supplemented with heat inactivated horse serum (5%) (Sigma), insulin (10 µg/ml) (Sigma), human epidermal growth factor (EGF) (20 ng/ml) (Peprotech), hydrocortisone (0.5 µg/ml) (Sigma) and 1% Pen/Strep (Invitrogen). Cells were sub-cultured regularly at 90% confluency. For the ATPS experiments, 5x10^5^ cells were seeded in each 35 mm^2^ Petri dishes in Pen/Strep-free medium. After 24 h, the cells formed a monolayer and were kept in Pen/Strep-free medium until the beginning of the experiment.

### Bacterial strains, plasmids and culturing conditions

The strains used in this study were *E. coli* MG1655, and *S. boydii* KACC 10792, which were obtained from the Korean RDA-Genebank Information Center (genebank.rda.go.kr), as well as P. sp DSM 50906 and *B* *. bacteriovorus* HD 100, both of which were purchased from the German Collection of Microorganisms and Cell Cultures (DSMZ).

*E. coli* MG1655 was transformed either with plasmid pAMCyan (Clontech, USA), which encodes for ampicillin resistance and the cyan fluorescent protein, with plasmid placCherry [10], which encodes for the cherry red fluorescent protein and provides kanamycin resistance, or with plasmid pHKT3 [43], which codes for the DsRed fluorescent protein and provides tetracycline resistance. Likewise, *P*. sp DSM 50906, and *S. boydii* KACC 10792 were both transformed with pHKT3.

All the bacterial strains were kept as frozen stocks in 20% glycerol at -80°C. *E. coli*, *S.* boydii, and P. sp strains were routinely streaked on LB agar plates, with ampicillin (100 µg/ml), kanamycin (50 µg/ml) or tetracycline (10 µg/ml) added, and incubated at 37°C for 24 h. After growth, a single colony was transferred to LB broth containing the appropriate antibiotic and incubated overnight in a shaking incubator at 37°C. This culture was used for the ATPS formulation.

For *B* *. bacteriovorus* HD 100, it was grown using a top agar plate prepared using dilute nutrient agar as described previously [38]. The top layer contained *E. coli* MG1655 as the prey. *B* *. bacteriovorus* growth was observed as a clear zone around the streak after 2 to 3 days. A small portion of this clear zone was aseptically transferred and then vortexed with 5 ml of DNB medium (1/10 dilution of nutrient broth containing 2 mM CaCl_2_ and 3 mM MgCl_2_). This medium was then filtered through a 0.45 µM filter to remove any remaining prey cells and agar while the predator, due to its small size, was able to pass through. A portion of this filtrate was then added to fresh prey cells as described below. The culture was then incubated in a shaking incubator at 30^°^C until the turbidity, i.e., the optical density, decreased and predator cells were clearly seen under a microscope. The optical density (OD) at 600 nm was measured using an Eppendorf Biospectrophotometer plus.

*B* *. bacteriovorus* HD 100 was then routinely sub-cultured every 12 h into 9 mL HEPES buffer (25 mM, pH 7.2) supplemented with 2 mM CaCl_2_ and 3 mM MgCl_2_. To prepare these subcultures, the prey (*E. coli* MG1655) was grown in LB broth overnight. From this culture, 1 ml was centrifuged and re-suspended in 9 ml of HEPES buffer. The 12 h old *B* *. bacteriovorus* HD 100 culture was filtered using a 0.45 µM syringe filter to remove any remaining prey and bdelloplasts and 1 ml of this was added to the 9 ml suspension of prey. These sub-cultures were then incubated again for 12 h more as before. This sub-culturing gave us on average 10^9^ predator cells/ml after growth. After approximately three weeks, the *B* *. bacteriovorus* HD 100 cultures were discarded and fresh cultures from stocks were prepared again.

### Preparation of ATPS solutions and bacterial suspensions

To prepare the aqueous two-phase system (ATPS) solutions, polyethylene glycol (PEG) (Sigma-Aldrich, Co.) and dextran (DEX) (Pharmacosmos, Denmark) were added to supplemented DMEM/F12 medium (without Pen/Strep) to give the required final concentrations. Unless mentioned otherwise, the M.W. of the PEG and DEX used were 35,000 and 500,000, respectively, while their final concentrations in the DMEM/F12 medium were 2.5% and 3.2% (w/w) respectively. After shaking the mixture thoroughly to dissolve the two polymers completely, the mixture was centrifuged at 4000 rpm and 4°C for 30 min, which yielded an upper PEG-rich phase and a lower DEX-rich phase. The two phases were then separated and sterilized using a 0.22 µm syringe filter. Both solutions were stored at 4°C until needed.

The DEX-rich phase was used to prepare the bacterial suspension to be spotted on the epithelial cell layer. Briefly, an overnight bacterial culture grown in LB broth was centrifuged at 16,000 x g for 2 minutes and the culturing medium discarded. The bacterial pellets were washed with and re-suspended in the DEX-rich DMEM/F12 solution to give the required reconstituted OD value (rOD). It should be noted here that, to ensure the bacterial spots remain well defined downstream, the LB medium should be removed as completely as possible before re-suspending the pellets in the DEX phase.

### Bacterial patterning using ATPS

After the MCF10a cells formed a homogenous nearly confluent layer (approximately 80% confluency when observed under an optical microscope) in 35mm plates, the medium was removed and the cells were washed with phosphate buffered saline (PBS) to remove any traces of antibiotics in the culture medium. After rinsing, 2.5 ml of the PEG-rich DMEM/F12 medium was added to the plate. The DEX-rich medium containing bacterial cells was then spotted using a micropipette as described previously [11]. Unless specified, the volume of the droplets was 0.3 µl, and the initial rOD of the bacterial spot was 0.05. After patterning, the plates were incubated at 37°C and 100% humidity for 24 h before analysis. A schematic diagram illustrating the process used for patterning is shown in Figure 1. Due to the low interfacial tension between the two phases, movement of the plates was done carefully and kept to a minimum.

### Predation of bacteria on the epithelial cells surface

*B* *. bacteriovorus* HD 100 was cultured as mentioned above for 12 h. From this culture, 1 ml was filtered using a 0.45 µm filter, centrifuged at 16,200 x g for 2 minutes and then re-suspended in 2.5 ml of the PEG-rich DMEM/F12 medium. This medium was then added to MCF10a monolayer plates as described before. For the control plates, 2.5 ml of the PEG-rich DMEM/F12 medium were added without *B* *. bacteriovorus* HD 100. Subsequently, the bacterial strains were patterned within DEX-rich droplets using the same protocol as above but at initial rOD of 0.5, unless noted otherwise.

### Microscopic analysis

The bacterial spots were observed using an inverted epifluorescence microscope (Olympus IX71) operated by Metamorph software and the images were captured with an Andor Luca 658 camera. Confocal microscopy was performed using an LSM700 confocal microscope (Carl Zeiss) operated by ZEN 2009 software.

To visualize the epithelial cells underneath the bacterial spots, Calcein AM dye (Life Technologies, USA) was used. Briefly, medium was removed from the culture and 1 mL PBS buffer containing the Calcein AM (1 µg/ml) was added to the Petri dish and incubated at 37^o^C for 30 minutes. Afterwards, the solution was removed and the plates were washed with PBS buffer alone, i.e., no dye, prior to visualizing the cells. When needed, dead epithelial cells were visualized simultaneously by adding Ethidium homodimer-1 (EthD-1, 0.004 mM) (Life Technologies, USA) into PBS buffer along with Calcein, AM dye.

To observe the bacterial community on the epithelial cells, medium removal and washing steps were performed gently to prevent any unnecessary damage to the adhered bacterial community. Quantitative analysis of the fluorescent images was done using ImageJ 1.42q software.

### Statistical analysis

Statistical analysis was done using GraphBad Prism program (version 5.01). Unless otherwise mentioned, experiments were performed in triplicate and the standard errors are presented by error bars on the graphs. For comparing two sets of data, t-test was used and statistical significance was pointed on the bar graphs using the marks (*, **, and ***) for *P* value < 0.05, 0.01, and 0.001 respectively. For comparing three or more sets of data, ANOVA test was performed, followed by Tukey test and statistically significantly different groups at *P* < 0.05 were designated with different letters (a, b, c, and d) on the bar graphs.

## Supporting Information

Figure S1 Comparing three different ATPS formulations for possible use for patterning bacteria on epithelial cells. MCF 10a cells were stained with calcein AM and EthD-1 (Live/Dead) stain and observed under epifluorescence microscope after 24 h of incubation with the PEG rich phase of each of the three formulations and the ordinary DMEM/F12 medium alone. A) Overlay images comparing the calcein AM and EthD-1 stained epithelial cells for each of the media preparations. Scale bar: 100 µm. B) Images showing the EthD-1 stained epithelial cells only. The large number of dead epithelial cells seen for the third formulation shows its toxic effect on MCF 10a cells compared to the other media preparations tested. C) ImageJ analysis for the images shown in Panel B. Y-axis shows the percentage area fluorescing red (dead epithelial cells) in each plate. 2 Petri dishes were prepared for each case with the error bars show the standard errors between them. Statistical analysis was performed using ANOVA followed by Tukey’ post hoc test (a, and b = *p* < 0.05). D) *E. coli* MG1655 spots formed on MCF10A cells using the first (left), and the second (right) ATPS formulations. *E. coli* MG1655 was transformed with pAmCyan which confers cyan fluorescence. The bacterial cells were spotted on MCF10A cells as 0.6 µl droplets and incubated for 24 h then the medium was removed and the plate was washed gently. The bacterial community formed using the 1^st^ formulation was robust and remained attached. In contrast, the bacterial community formed using the 2^nd^ formulation was weakly attaching to the underlying epithelial cells and most of it was removed upon washing the plate. Scale bar: 1mm.

Figure S2 Comparing ATPS culturing with the ordinary culturing of bacteria on polystyrene surface. *E. coli* MG1655 was spotted in plain 35 mm Petri dishes (with no epithelial cells cultured), at different rOD values using both ordinary culturing and the ATPS technique. For the former, the bacteria were resuspended into ordinary DMEM/F12 medium at the required rOD and then spotted directly as single 0.3 µl droplet into 2.5 ml of the medium. For the ATPS patterning, the bacteria were resuspended in DEX rich DMEM/F12 medium and one 0.3 µl droplet was spotted in 2.5 ml of PEG rich medium. All plates were then incubated at 37°C for 24 h. The media were then aspirated into 10 ml glass tubes and their pHs were measured. The error bars represent the standard errors of three replicates for each case. Statistical analysis was performed using ANOVA followed by Tukey post hoc test (a, b, c, d and e = *p* < 0.05).

Figure S3 The macroscopical appearance of ATPS derived *E. coli*, *S.* boydii, and *P*. sp bacterial communities on MCF 10a monolayer. The three strains were spotted as 0.3 µl droplets on a single MCF 10a monolayer at an initial rOD of 0.5 in DMEM/F12 medium. *E. coli* MG1655 and *S. boydii* KACC 10792 formed clearly visible bacterial communities after 24 h of incubation at 37°C. In contrast, P. sp DSM 50906 grew slowly under these conditions and its spot could only be seen using microscopy.

Figure S4 The microscopical appearance of the epithelial layer underneath *E. coli* and *S. boydii* ATPS derived spots. *E. coli* MG1655 and *S. boydii* KACC 10792 ATPS derived communities were not harmful to the underlying MCF 10a cells. This is clear from the integrity and well being of the epithelial cell monolayer underneath each of these two bacterial spots. Images were taken using an epifluorescence microscope. Scale bar: 500 µm

Figure S5 Effect of initial rOD of *P*. sp on the damage caused to the underlying MCF 10a cells layer. The central photo shows the P. sp DSM 50906 communities made using four different initial rOD values (5, 0.5, 0.05, and 0.005) all were spotted as 0.3 µl droplets on the MCF 10a monolayer in the same plate. The analysis was done after 24 h of incubation at 37°C. The peripheral images show the underlying epithelial layer for each spot while the central image shows the four bacterial communities with the underlying epithelial sheet. The peripheral images were taken using an epifluorescence microscope while the central one was obtained by tile scanning using a confocal microscope (scale bar: 1mm).
