# Supplementary figures and images for: Patterning Bacterial Communities on Epithelial Cells
Source: PLoS One. 2013 Jun 13;8(6):e67165. doi: 10.1371/journal.pone.0067165 (PMC3681762; doi:10.1371/journal.pone.0067165)

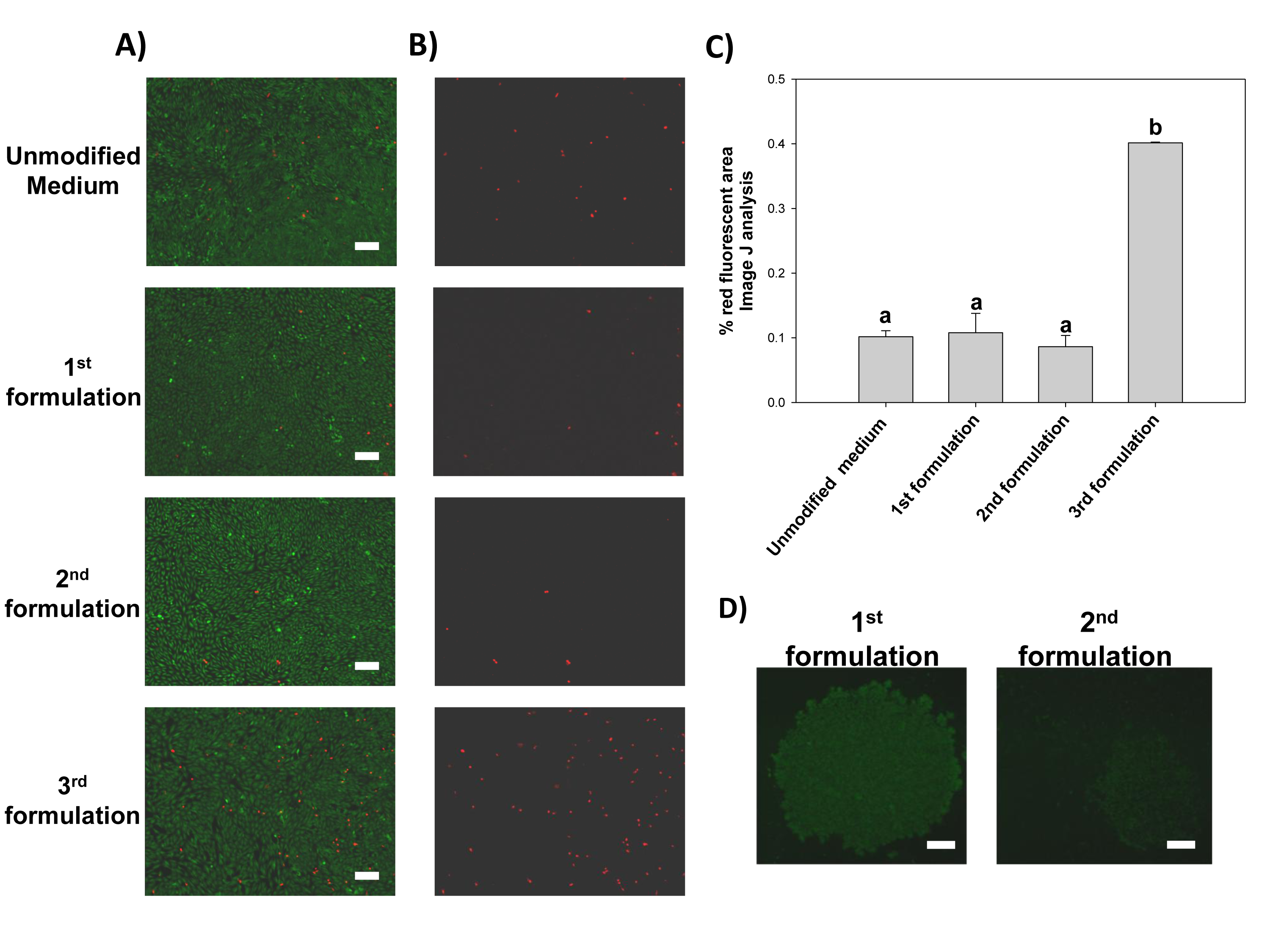

Supplement: Figure S1 — MCF 10a cells were stained with calcein AM and EthD-1 (Live/Dead) stain and observed under epifluorescence microscope after 24 h of incubation with the PEG rich phase of each of the three formulations and the ordinary DMEM/F12 medium alone. A) Overlay images comparing the calcein AM and EthD-1 stained epithelial cells for each of the media preparations. Scale bar: 100 µm. B) Images showing the EthD-1 stained epithelial cells only. The large number of dead epithelial cells seen for the third formulation shows its toxic effect on MCF 10a cells compared to the other media preparations tested. C) ImageJ analysis for the images shown in Panel B. Y-axis shows the percentage area fluorescing red (dead epithelial cells) in each plate. 2 Petri dishes were prepared for each case with the error bars show the standard errors between them. Statistical analysis was performed using ANOVA followed by Tukey’ post hoc test (a, and b = p < 0.05). D) E. coli MG1655 spots formed on MCF10A cells using the first (left), and the second (right) ATPS formulations. E. coli MG1655 was transformed with pAmCyan which confers cyan fluorescence. The bacterial cells were spotted on MCF10A cells as 0.6 µl droplets and incubated for 24 h then the medium was removed and the plate was washed gently. The bacterial community formed using the 1st formulation was robust and remained attached. In contrast, the bacterial community formed using the 2nd formulation was weakly attaching to the underlying epithelial cells and most of it was removed upon washing the plate. Scale bar: 1mm. [file pone.0067165.s001.tif]

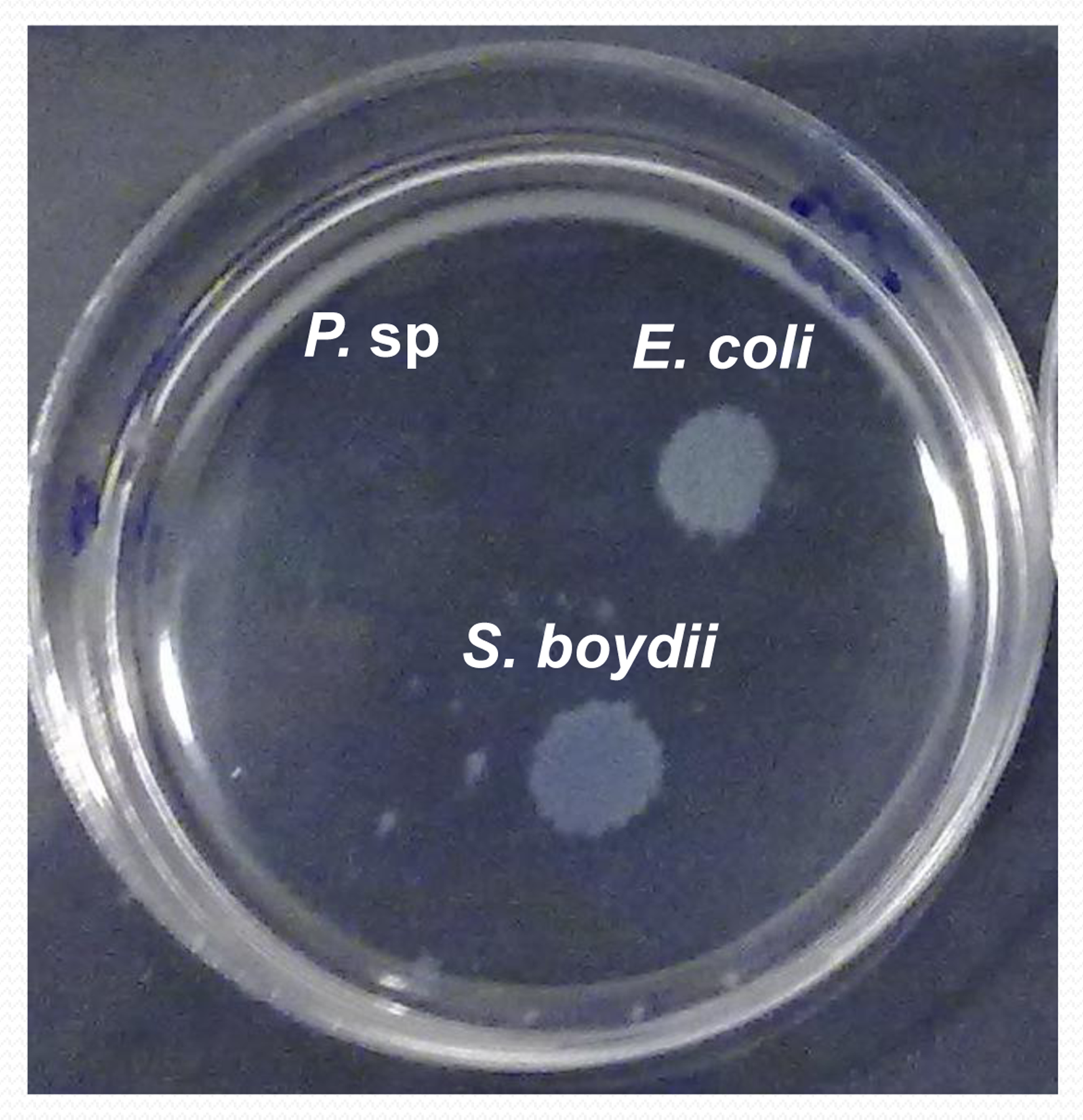

Supplement: Figure S3 — The three strains were spotted as 0.3 µl droplets on a single MCF 10a monolayer at an initial rOD of 0.5 in DMEM/F12 medium. E. coli MG1655 and S. boydii KACC 10792 formed clearly visible bacterial communities after 24 h of incubation at 37°C. In contrast, P. sp DSM 50906 grew slowly under these conditions and its spot could only be seen using microscopy. [file pone.0067165.s003.tif]

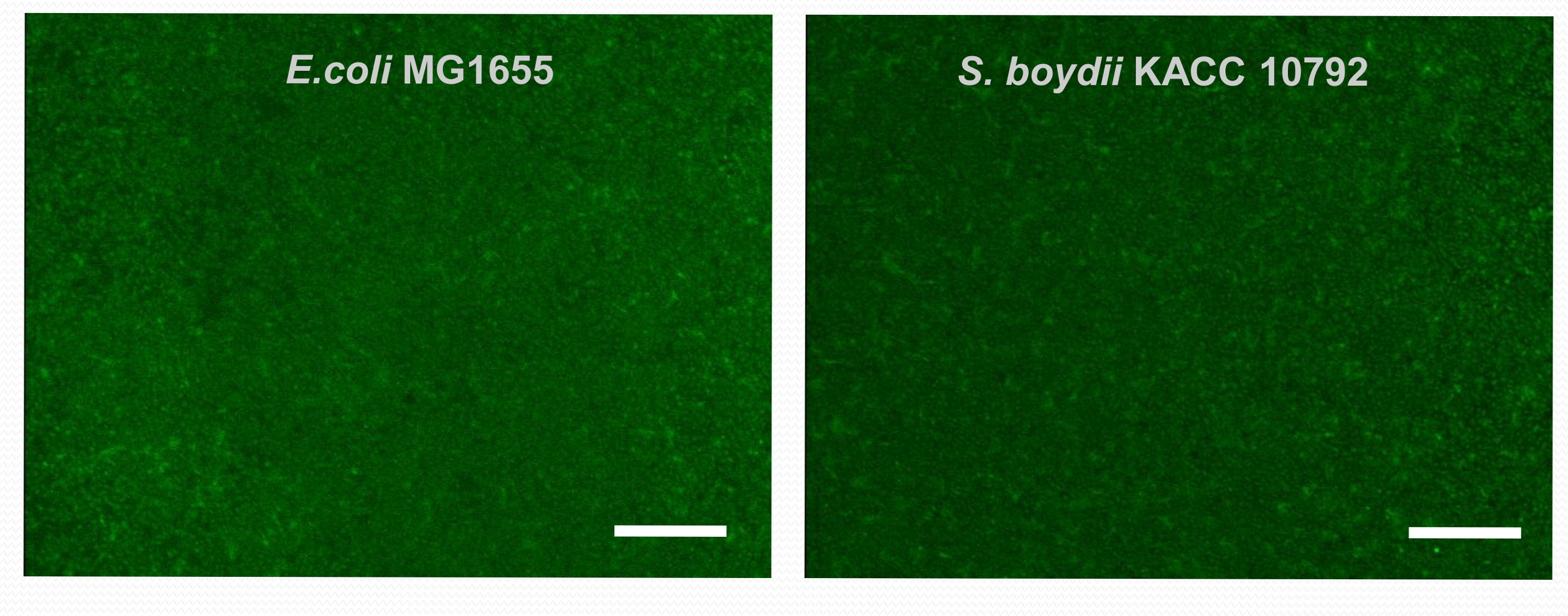

Supplement: Figure S4 — E. coli MG1655 and S. boydii KACC 10792 ATPS derived communities were not harmful to the underlying MCF 10a cells. This is clear from the integrity and well being of the epithelial cell monolayer underneath each of these two bacterial spots. Images were taken using an epifluorescence microscope. Scale bar: 500 µm [file pone.0067165.s004.tif]

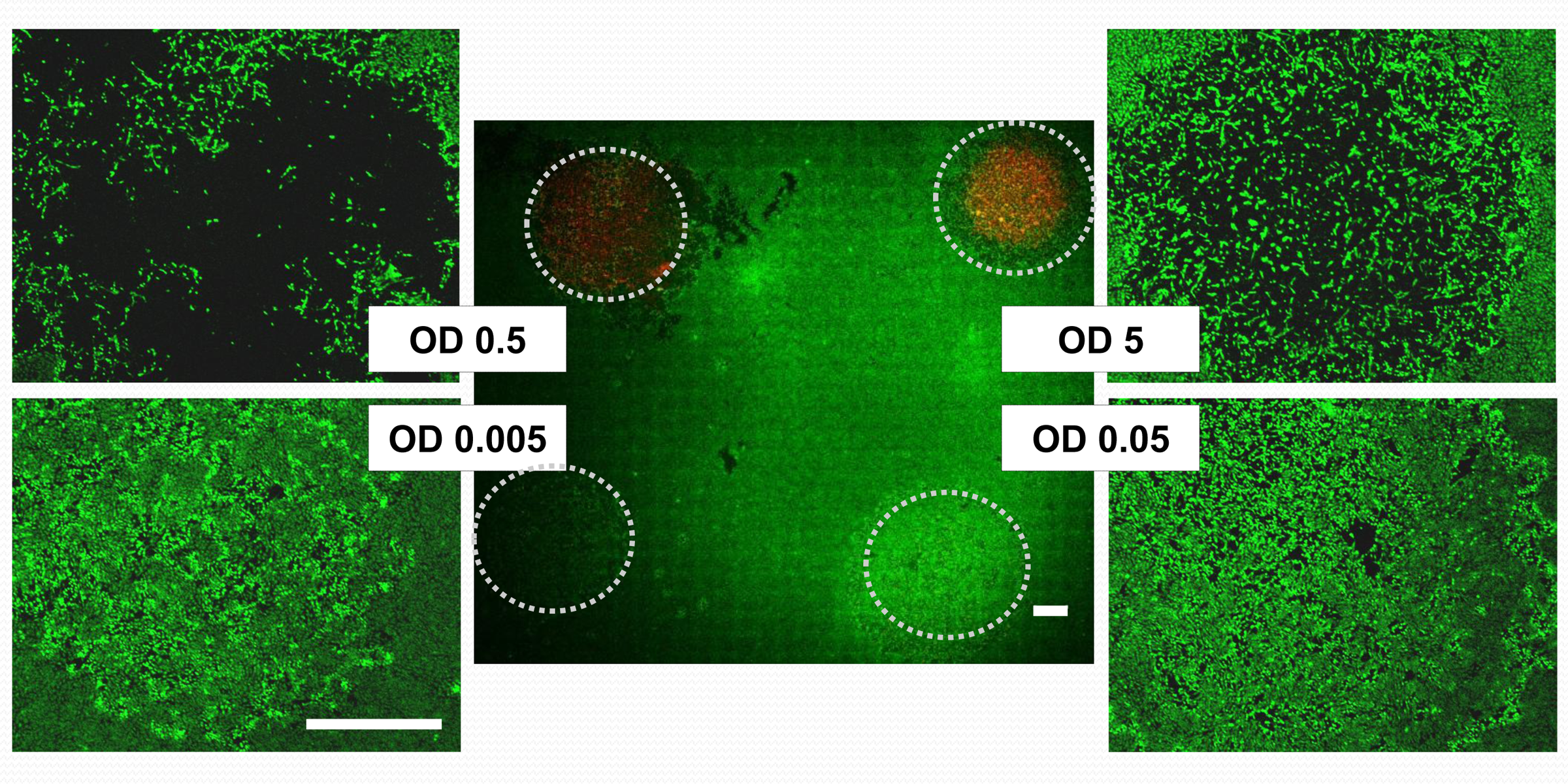

Supplement: Figure S5 — The central photo shows the P. sp DSM 50906 communities made using four different initial rOD values (5, 0.5, 0.05, and 0.005) all were spotted as 0.3 µl droplets on the MCF 10a monolayer in the same plate. The analysis was done after 24 h of incubation at 37°C. The peripheral images show the underlying epithelial layer for each spot while the central image shows the four bacterial communities with the underlying epithelial sheet. The peripheral images were taken using an epifluorescence microscope while the central one was obtained by tile scanning using a confocal microscope (scale bar: 1mm). [file pone.0067165.s005.tif]
